# Adequacy of Lateral Ankle Radiograph Views in Acute Trauma Setting: A Quality Improvement Project

**DOI:** 10.7759/cureus.91479

**Published:** 2025-09-02

**Authors:** Hassan Imtiaz, Adam Rahim, Haider Paracha, Saqib Samad, Georgios Kouklidis, Antony Raymond

**Affiliations:** 1 Trauma and Orthopaedics, University Hospitals Dorset, Poole, GBR; 2 Orthopaedics, University Hospitals Dorset, Poole, GBR

**Keywords:** ankle and foot, general radiology, lateral radiograph, orthopaedics trauma, quality improvement (qi)

## Abstract

Introduction: Accurate radiographic assessment is critical in the management of ankle trauma, where lateral ankle views play a key role in identifying fractures and assessing joint alignment. Inadequate radiographs can lead to diagnostic delays or mismanagement. This quality improvement project aimed to evaluate and improve the adequacy of lateral ankle radiographs in a trauma setting, in line with British Orthopaedic Association Standards for Trauma (BOAST) guidelines.

Methods: A retrospective closed-loop audit was conducted in a UK trauma unit. One hundred lateral ankle radiographs were evaluated in the first cycle based on four criteria for a "true lateral" view: (1) distal fibula superimposed by posterior tibia; (2) talar domes superimposed; (3) open and uniform tibiotalar joint space; and (4) inclusion of anatomical landmarks (lower third of tibia and fibula, talus, calcaneus, and base of the fifth metatarsal). Following the first cycle, targeted interventions, educational sessions, and visual reference posters were implemented. A second cycle of 100 radiographs was then assessed using the same criteria. Statistical comparison between the two cycles was performed using the chi-squared test.

Results: In the first cycle, compliance rates were 83% (criterion one), 61% (criterion two), 69% (criterion three), and 99% (criterion four). After the intervention, the second cycle showed improvements across criteria one to three, with compliance rates of 95%, 85%, and 92%, respectively. Criterion four remained unchanged at 99%. The chi-squared analysis demonstrated statistically significant improvements for criteria one (p=0.0129), two (p=0.0002), and three (p=0.0001), but not for criterion four (p=1.0).

Conclusion: This audit demonstrated that simple, cost-effective educational interventions significantly improved the adequacy of lateral ankle radiographs in a trauma setting. Sustained improvement requires continued reinforcement of radiographic standards, multidisciplinary collaboration, and routine audit cycles to ensure consistent image quality in acute care.

## Introduction

Ankle injuries are among the most prevalent musculoskeletal complaints presenting to emergency departments, including ankle sprains, fractures, dislocations or subluxations, and ligament injuries. These account for approximately 5% of all emergency visits in the United Kingdom [1]. Of these, ankle fractures represent around 9% of all fractures seen in trauma services, with a high incidence among young adults engaged in sports and older adults with osteoporosis [2,3]. Accurate and timely diagnosis is essential for appropriate management, and plain radiography remains the first-line imaging modality for suspected fractures in acute settings [4].

In particular, the lateral view of the ankle is crucial for assessing fracture alignment, joint congruity, and soft tissue involvement. It facilitates evaluation of posterior malleolar fractures, talar dome congruency, and adjacent anatomical landmarks such as the subtalar joint and base of the fifth metatarsal [5]. However, obtaining a technically adequate true lateral view can be challenging in trauma settings due to patient discomfort, limited mobility, or deformity, often resulting in suboptimal imaging [6].

The British Orthopaedic Association Standards for Trauma (BOAST) recommend that a true lateral ankle radiograph should be centered on the ankle joint and include superimposed talar domes, partial superimposition of the distal fibula over the posterior tibia, and capture of the lower third of the tibia and fibula, the talus, the calcaneus, and the base of the fifth metatarsal [7]. Substandard images can lead to missed diagnoses, delayed care, and increased costs [8]. This project aimed to assess the adequacy of lateral ankle radiographs in a busy trauma unit, implement targeted interventions, and evaluate their effectiveness in a closed-loop audit.

## Materials and methods

This is a retrospective, closed-loop audit conducted at a trauma unit at a district general hospital in the United Kingdom. As per the NHS Health Research Authority online decision tool [9], this study was not considered research and did not require ethical approval. The audit was registered locally with the hospital clinical governance team. The first audit cycle evaluated data from the dates between 01/09/2023 and 31/12/2023. Gaps in practice were identified and presented at the local clinical governance meeting, followed by an agreement to target improved compliance with the set standards by means of putting up posters in relevant clinical areas, such as emergency departments and the orthopaedic on-call room, to serve as a visual aid to remind teams to ensure that the key elements of a true lateral radiograph are met. A dedicated teaching session by an associate specialist foot and ankle surgeon (see Appendix A) was conducted for orthopaedic team members and radiographers, focusing on the importance of accurate lateral ankle radiographs, ensuring appropriate positioning to get the desired view, and key landmarks to look for in these views. Following the aforementioned educational interventions, the subsequent audit cycle was conducted, evaluating data between 01/03/2024 and 30/06/2024. 

Audit standards were derived from BOAST guidelines [7] and radiographic positioning literature based on Clark’s position in radiography [10] and the online resource, Radiopedia [11]. These are presented in Table 1. Target compliance was set at 100% for each standard following agreement between the orthopaedic and radiology departments. 

**Table 1:** Audit standards

Audit standard(s)	Target compliance (%)
Criteria 1: Distal fibula superimposed by posterior tibia	100
Criteria 2: Talar domes superimposed	100
Criteria 3: Open and uniform tibiotalar joint space	100
Criteria 4: Inclusion of key anatomical landmarks (lower tibia/fibula, talus, calcaneus, base of 5th metatarsal)	100

Patients presenting with acute ankle trauma were identified from e-trauma (a digital platform for managing orthopaedic trauma in the NHS to improve coordination and workflow) and theatre list records taken from the trust intranet. Patients aged 18 years and above presenting with acute ankle trauma were included in the audit. Those with previous ankle surgery, open fractures, or fracture dislocations of the ankle, the presence of a cast, or those undergoing weight-bearing lateral views met the exclusion criteria. 

Consecutive eligible lateral ankle radiographs in the given time frames were evaluated on the Picture Archiving and Communication System (PACS) imaging viewer and assessed independently by a senior radiologist and a foot and ankle associate specialist surgeon to assess whether each of the audit standards was met for each radiograph. In cases of conflict among decisions on a parameter, a third opinion was sought from a consultant radiologist. 

Data was collected, stored, and analysed using Microsoft Excel (Microsoft Corp., Redmond, WA, USA). Patient identifiers were removed from the datasheet before final analysis. The chi-squared test was employed to statistically compare the results of the first and second loop results, with a p-value of less than 0.05 being set as the mark for statistical significance. 

## Results

A total of 100 radiographs that met the inclusion criteria were assessed for both audit cycles. For the first audit cycle, 83% (n=83) of radiographs showed appropriate superimposition of the distal fibula by the posterior tibia (criterion one). Only 61% (n=61) demonstrated adequate talar dome superimposition (criterion two), and 69% (n=69) exhibited a clearly open and uniform tibiotalar joint space (criterion three). Criterion four, which assesses whether the lateral view includes the required anatomical landmarks, the lower third of the tibia and fibula, talus, calcaneus, and base of the fifth metatarsal, had the highest compliance at 99% (n=99). The most common reason for failure to meet criterion four was omission of the base of the fifth metatarsal in the field of view. None of the cases required a consultant radiologist’s opinion to clarify ambiguities. These results are presented in Table 2. 

**Table 2:** First audit cycle results

Audit standard(s)	Observed compliance (%)	Target compliance (%)
Criteria 1: Distal fibula superimposed by posterior tibia	83	100
Criteria 2: Talar domes superimposed	61	100
Criteria 3: Open and uniform tibiotalar joint space	69	100
Criteria 4: Inclusion of key anatomical landmarks (lower tibia/fibula, talus, calcaneus, base of 5th metatarsal)	99	100

Following the implementation of targeted interventions, namely an educational session for radiographers and the placement of standardised positioning posters in radiographic suites, a second audit cycle of 100 radiographs was conducted. This re-audit demonstrated notable improvement across most domains. Compliance for criterion one increased to 95% (n=95), while criterion two improved to 85% (n=85). Criterion three saw a rise to 92% (n=92), reflecting a more consistent achievement of uniform tibiotalar joint space visualization. Criterion four remained unchanged at 99% (n=99), as it had already achieved near-complete compliance in the first cycle. None of the cases required a consultant radiologist’s opinion to clarify ambiguities. The results of the second audit cycle are presented in Table 3. 

**Table 3:** Second audit cycle results

Audit standard(s)	Observed compliance (%)	Target compliance (%)
Criteria 1: Distal fibula superimposed by posterior tibia	95	100
Criteria 2: Talar domes superimposed	85	100
Criteria 3: Open and uniform tibiotalar joint space	92	100
Criteria 4: Inclusion of key anatomical landmarks (lower tibia/fibula, talus, calcaneus, base of 5th metatarsal)	99	100

When comparing the first and second audit loops using chi-squared statistical analysis, significant improvements were observed in criteria one, two, and three. For criterion one, the increase from 83% to 95% yielded a p-value of 0.0129, indicating statistical significance. Similarly, the improvement in criterion two, from 61% to 85%, was highly significant (p=0.0002). Criterion three also showed a statistically significant increase from 69% to 92% (p=0.0001). In contrast, the minimal change in criterion four compliance (99% in both cycles) was not statistically significant (p=1.0000). The comparison between audit cycles is presented in Table 4. 

**Table 4:** Statistical analysis *Chi-squared Test used for assessing statistical significance; **A p-value of < 0.05 set as measure of statistical significance

Audit standard(s)	p-value*	Statistically significant** (yes/no)
Criteria 1: Distal fibula superimposed by posterior tibia	0.0129	Yes
Criteria 2: Talar domes superimposed	0.0002	Yes
Criteria 3: Open and uniform tibiotalar joint space	0.0001	Yes
Criteria 4: Inclusion of key anatomical landmarks (lower tibia/fibula, talus, calcaneus, base of 5th metatarsal)	1.0	No

## Discussion

This quality improvement audit highlighted substantial initial deficiencies in the adequacy of lateral ankle radiographs taken in a trauma unit. The first audit cycle showed variability in meeting key imaging criteria, with compliance for talar dome superimposition as low as 61%. 

Lateral ankle views are critical in detecting subtle posterior malleolar or talar dome fractures, and poor-quality imaging can contribute to underdiagnosis or inappropriate management [12,13]. Although advanced imaging such as CT or MRI may offer higher diagnostic accuracy, plain radiography remains the most accessible and widely used modality in initial trauma evaluation [13].

After introducing targeted educational interventions, compliance improved significantly across all parameters. Criteria one to three improved to 95%, 85%, and 92%, respectively, indicating that structured staff education and visible reminders can enhance image quality. Nevertheless, criterion two (talar dome superimposition) remained the most challenging to meet, reflecting the technical difficulty of achieving true lateral alignment in a trauma setting. This can result in misinterpretations of ankle radiographs [14].

Adequate lateral ankle views can provide crucial measurements such as the posterior tibial line, Bohler angle, angle of Gissane, anterior to posterior fibular gap, and lateral tibiotalar distance, thus guiding clinicians in choosing optimal treatment strategies [15]. A review of the literature reveals that ankle fractures are among the most commonly misinterpreted acute injuries presenting to the emergency department [16,17]. A study by Guo et al. reported significant disagreement regarding inter-observer reliability among foot and ankle surgeons evaluating an incongruent ankle joint (for cases where the distance between the central talar and distal tibia articular surface distance was < 4 millimeters) based on lateral ankle radiographs [18], which further reinforces the importance of ensuring an adequate lateral ankle view. The use of a closed-loop audit cycle proved effective in assessing the impact of interventions, reinforcing previous findings that continuous feedback and multidisciplinary engagement improve adherence to standards [19]. Long-term sustainability could be improved by integrating feedback into routine practice.

While this study demonstrates encouraging results, limitations must be acknowledged. First, the sample size, though adequate for initial quality assessment, was limited to a single center and cannot indicate variations among other institutes. Secondly, the audit did not assess clinical outcomes such as changes in management or diagnostic accuracy, which would further clarify the clinical impact of lateral ankle radiograph adequacy. Lastly, the long-term sustainability of improved compliance was not evaluated, which could also highlight the reliability of proposed interventions for targeting optimal performance. 

## Conclusions

This audit demonstrated that simple, cost-effective educational interventions significantly improved the adequacy of lateral ankle radiographs in a trauma setting. The improvements observed were statistically significant, particularly in achieving better visualization of the talar domes and tibiotalar joint space. Given the pivotal role of lateral ankle radiographs in initial trauma assessment and the potential for misdiagnosis when images are inadequate, radiographic quality must be prioritized alongside clinical acumen. Sustained improvement requires continued reinforcement of radiographic standards, multidisciplinary collaboration, and routine audit cycles to ensure consistent image quality in acute care.

## Figures and Tables

**Figure 1:**
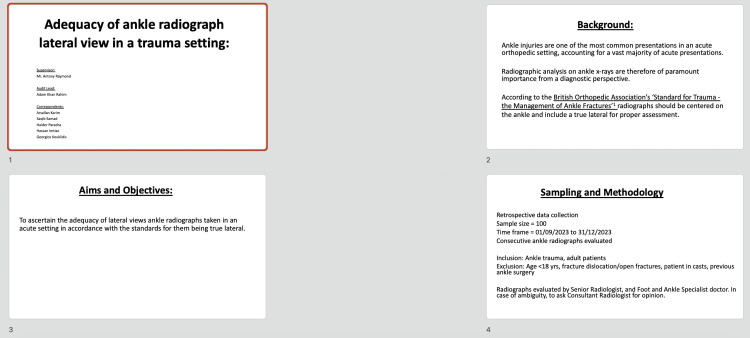
Slides 1 to 4 of the PowerPoint presentation

**Figure 2:**
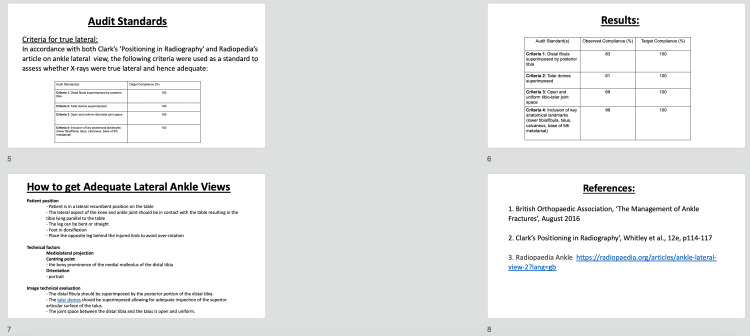
Slides 5 to 8 of the PowerPoint presentation

## Appendices

Appendix A

Figures 1-2 feature PowerPoint (Microsoft Corp., Redmond, WA, USA) slides used in the teaching session conducted by an associate specialist foot and ankle surgeon after the first audit cycle's results. These slides highlight the areas of suboptimal compliance and focus on how to obtain adequate lateral ankle radiographs. 
